# The end of fragmented consciousness: deaths in schizophrenia—an autopsy-based study

**DOI:** 10.1007/s11845-025-04274-y

**Published:** 2026-01-19

**Authors:** Filiz Ekim Çevi̇k, Aytül Buğra, Muhammed Oduncu, Yasin Kavla, Hüseyin Çağrı Şahi̇n, Muhammed Fatih Yaman, Furkan Doğan, Hatice Büşra Arisin, İbrahim Üzün, Hızır Asliyüksek

**Affiliations:** 1 Department of Medical Sciences, Institute of Forensic Sciences and Legal Medicine, Istanbul University-Cerrahpasa, Istanbul, Türkiye; 2 Council of Forensic Medicine, Istanbul, Türkiye; 3 Department of Psychiatry, Cerrahpasa Medical Faculty, Istanbul University-Cerrahpasa, Istanbul, Türkiye; 4 Department of Public Health, Marmara Medical Faculty, Marmara University, Istanbul, Türkiye

**Keywords:** Autopsy, Death, Forensic autopsy, Schizophrenia, Toxicology

## Abstract

**Background:**

Deaths in schizophrenia are multifactorial, yet postmortem data from Türkiye are limited. This study evaluated demographic, clinical, and postmortem characteristics of patients with schizophrenia, with particular focus on antipsychotic use, death circumstances, and organ pathology.

**Method:**

A retrospective analysis was conducted on 107 autopsy cases from 2020 to 2024. Data on demographics, clinical history, toxicology, and macroscopic organ findings were collected and analyzed.

**Results:**

Most patients were male (70.1%) with a mean age of 50 ± 13.8 years. Natural deaths accounted for 56.1%, predominantly cardiovascular, whereas non-natural deaths comprised 43.9%, mainly trauma and asphyxia. Antipsychotics were detected in 61.3% of cases, often in combination, and antidepressants in 21.7%. Combined antipsychotic use was associated with a lower likelihood of being found dead.

**Conclusion:**

Deaths in schizophrenia are influenced by cardiovascular, traumatic, metabolic, and pharmacological factors. Postmortem evaluations indicate limited macroscopic effects of psychotropic medications. Mortality risk assessment should integrate clinical, pharmacological, and sociodemographic factors.

## Introduction

Schizophrenia is a mental disorder associated with a substantially increased risk of mortality compared to the general population, characterized by positive symptoms such as delusions and hallucinations, and negative symptoms including lack of motivation and social withdrawal [1, 2]. Life expectancy in patients with schizophrenia is approximately 15–25 years shorter than in the general population, with higher rates of sudden death [3, 4]. In a 10-year Australian autopsy-based study involving 683 cases with a history of schizophrenia, the mean age at death was 51 years (range 18–93), with nearly half of deaths (43%) occurring in middle age (41–60 years) and over two-thirds (67%) among males [5]. Studies in psychiatric clinics report a sudden death rate of 0.8%, markedly higher than the 0.1–0.2% observed in the general population [6].

Sudden death in schizophrenia is multifactorial. Autopsy studies indicate that cardiovascular diseases are the leading cause of death, accounting for approximately 50% of cases, while metabolic syndrome prevalence exceeds 35% in men and 50% in women [6, 7]. Smoking rates in this population range from 66 to 73%, significantly higher than in the general population (23–35%) [8]. Cardiac side effects of antipsychotics also contribute to sudden death risk. Clozapine, although the gold standard for treatment-resistant schizophrenia, has been associated with myocarditis and sudden cardiac death, likely through QT interval prolongation, myocarditis, and hypersensitivity reactions [9–11]. Pneumonia and pulmonary thromboembolism are less frequent causes, while some sudden deaths remain unexplained, presumably due to cardiac arrhythmias [5, 12].

Schizophrenia is increasingly recognized as a systemic disorder with multiple somatic burdens. Pathophysiological processes—including elevated cortisol levels, autonomic dysfunction, inflammation, lipid metabolism abnormalities, increased oxidative stress, and enhanced platelet reactivity—contribute to cardiovascular disease development and progression, thereby increasing sudden cardiac death risk [13].

Several autopsy-based studies on sudden unexplained death (SUD) in schizophrenia have been reported from China [14], the United States [15, 16], the United Kingdom [17], Romania [6], and Australia [5]. Autopsy studies are critical for understanding the pathogenesis of sudden death, allowing identification of organ-specific pathologies, drug effects, and comorbid risk factors.

To investigate the situation in Türkiye and clarify potential risk factors, we retrospectively analyzed deaths in schizophrenia patients autopsied at the Istanbul Specialized Morgue Department between 2020 and 2024. Information on the timing and location of death, antipsychotic use, and autopsy findings was collected to provide a systematic forensic analysis of pathological and toxicological features, contribute to current literature, and guide future research.

## Materials and methods

### Study population

All cases included in this study had a prior diagnosis of schizophrenia according to DSM-5 criteria [18]. The study was approved by the Council of Forensic Medicine, Education and Scientific Research Commission (21589509/2025/373 − 18/03/2025). Medical records of schizophrenia patients autopsied at the Council of Forensic Medicine Morgue Specialty Department between 2020 and 2024 were retrospectively reviewed. Pathologies contributing to death, along with autopsy and histopathological findings, were analyzed. Clinical histories, next-of-kin information, comorbidities, and toxicology results were extracted to identify demographic and pathological factors associated with sudden death. Treatment adherence was assessed by comparing prescribed medications in medical records with drugs detected in postmortem blood samples. The association between antipsychotic use and organ-specific pathological findings was also evaluated.

### Statistical analysis

Statistical analyses were performed using IBM SPSS Statistics 25.0. Categorical variables are presented as counts (n) and percentages (%), while continuous variables are presented as mean ± standard deviation (SD). Categorical variables were compared using Pearson’s Chi-square (χ²) test or Fisher’s Exact test, depending on expected cell frequencies; Fisher’s Exact test was applied when expected counts were less than five. Data distribution for continuous variables was assessed using histograms and the Kolmogorov-Smirnov test. Depending on distribution, group comparisons were performed using the Independent Samples t-test for parametric data or the Mann-Whitney U test for non-parametric data. A p-value < 0.05 was considered statistically significant.

## Results

This study included 107 cases with a clinical history of schizophrenia who underwent autopsy at the Istanbul Specialized Morgue Department between 2020 and 2024. Of these, 70.1% (*n* = 75) were male and 29.9% (*n* = 32) were female. The mean age of all cases was 49.85 ± 13.75 years (range, 17–76), with women having a mean age of 51.03 ± 14.26 years and men 49.35 ± 13.59 years; the difference was not statistically significant (*p* > 0.05).

Regarding circumstances of death, 51.4% (*n* = 55) were found deceased, 37.4% (*n* = 40) experienced sudden illness, and 11.2% (*n* = 12) died following trauma. Deaths occurred in open areas in 50.5% (*n* = 54) and in enclosed spaces in 49.5% (*n* = 53).

Analysis of identity witnesses reported by prosecutors showed that 60.7% (*n* = 65) were first-degree relatives, 26.2% (*n* = 28) were other relatives or friends, 6.5% (*n* = 7) were other individuals, and 6.5% (*n* = 7) had no witnesses. Medical intervention prior to death was performed in 46.7% (*n* = 50) of cases. Tattoos were observed on external examination in 8.4% (*n* = 9) of cases (Table 1).

**Table 1:** Sociodemographic and clinical characteristics

		*n*	%
Gender	Male	75	70.1
	Female	32	29.9
Circumstance of Death	Found dead	55	51.4
	Sudden illness	40	37.4
	Traumatic	12	11.2
Place of Death	Indoor	53	49.5
	Outdoor	54	50.5
Identity Witness	First-Degree Family	65	60.7
	Relative-Friend	28	26.2
	Other	7	6.5
	None	7	6.5
Medical Intervention	Present	50	46.7
	Absent	57	53.3
Tattoo	Present	9	8.4
	Absent	98	91.6

Organ weights recorded during autopsy showed a mean heart weight of 360.00 ± 118 g, mean lung weight of 1198.26 ± 547.22 g, and mean brain weight of 1288.00 ± 215 g.

External and internal examinations revealed skin decomposition in 30.8% (*n* = 33) of cases; brain atrophy in 19.6% (*n* = 20); myocardial lesions due to prior ischemic injury in 28.2% (*n* = 29); cardiac hypertrophy in 19.4% (*n* = 20); pleural petechial hemorrhage in 21.9% (*n* = 23); and yellowish liver discoloration in 36.8% (*n* = 39) (Table 2). Decomposition was observed in 60% (*n* = 33) of cases that were found deceased.

**Table 2:** Macroscopic findings of organs

Organ	Findings		*n*	%
Skin	Signs of Putrefaction	Present	33	30.8
		Absent	74	69.2
Brain	Brain Atrophy	Present	20	19.6
		Absent	82	80.4
Heart	Cardiac Hypertrophy	Present	20	19.4
		Absent	83	80.6
	Ischemic Lesion	Present	29	28.2
		Absent	74	71.8
Lungs	Pleural Petechial Hemorrhage	Present	23	21.9
		Absent	82	78.1
	Tracheobronchial Obstructive Lesion	Present	1	0.9
		Absent	105	99.1
Liver	Yellowish Color Change	Present	39	36.8
		Absent	67	63.2
	Nodular Micronodular Cirrhosis	Present	1	0.9
		Absent	105	99.1

Cause of death analysis indicated that 56.1% (*n* = 60) of cases were natural deaths, while 43.9% (*n* = 47) were unnatural. Among natural deaths, cardiovascular causes were most common (58.3%, *n* = 35), followed by pneumonia (21.7%, *n* = 13), other infections (10%, *n* = 6), cancer (6.7%, *n* = 4), and epilepsy (3.3%, *n* = 2). Among unnatural deaths, blunt trauma was the most frequent (26.7%, *n* = 8), followed by poisoning (*n* = 7), asphyxia (*n* = 7), drowning (*n* = 3), sharp force injury (*n* = 2), fire exposure (*n* = 2), and traffic injuries (*n* = 1). The cause of death remained undetermined in 17 cases (Table 3).

**Table 3:** Causes of death in schizophrenia cases

Cause of Death		*n*	%
Natural Deaths ***Total***	Cardiovascular	35	58.3
	Pneumonia	13	21.7
	Infection	6	10.0
	Cancer	4	6.7
	Epilepsy	2	3.3
		60	%100
Unnatural Deaths ***Total***	Traffic Injury	1	3.3
	Blunt Trauma	8	26.7
	Sharp Force Injury	2	6.7
	Asphyxia	7	23.3
	Burn	2	6.7
	Drowning	3	10.0
	Poisoning	7	23.3
		30	%100
Unknown		17	

Comparison of cases resulting in natural versus unnatural death showed no significant differences between genders. Evaluation by event type indicated that natural deaths were significantly more frequent in cases of sudden illness, whereas unnatural deaths were significantly more frequent in trauma-related cases (*p* = 0.008 and *p* < 0.001, respectively). The presence of skin decomposition was significantly associated with unnatural deaths (*p* = 0.023). No significant differences were observed between groups regarding antidepressant use. When body mass index (BMI) was categorized as below or above 25, cases with BMI < 25 were significantly more frequent among natural deaths compared to unnatural deaths (*p* = 0.033) (Table 4).

**Table 4:** Clinical characteristics of natural and unnatural deaths

	Natural		Unnatural		
	*n*(60)	%	*n*(47)	%	*p*
Gender					
Female	18	56.2	14	43.8	1.00
Male	42	56.0	33	44.0	
Circumstance of Death					
Found dead	30	54.5	25	45.5	< 0.001*
Sudden illness	29	72.5	11	27.5	
Traumatic	1	8.3	11	91.7	
Signs of Putrefaction					
Present	13	39.4	20	60.6	0.023*
Absent	47	63.5	27	36.5	
Antidepressant Use					
Yes	9	39.1	14	60.9	0.062
No	51	61.4	32	38.6	
BMI					
< 25	36	66.7	18	33.3	0.033*
≥ 25	24	45.3	29	54.7	

* *p* < 0.05 was considered significant

Toxicological analysis of postmortem blood samples revealed antipsychotic drugs in 61.3% (*n* = 65) of cases. Among cases with antidepressants detected in postmortem blood, 39.1% (*n* = 9) were natural deaths and 60.9% (*n* = 14) were unnatural deaths. Combined antipsychotic use was observed in 50% (*n* = 53) of cases. Among the detected antipsychotics, quetiapine was the most frequent (64.5%), followed by risperidone (32.7%), paliperidone (18.7%), amisulpride (18.7%), and zuclopenthixol (18.7%) (Table 5).

**Table 5:** Antipsychotics detected according to postmortem toxicology results

Antipsychotics	*n*	%
Olanzapine	14	13.1
Risperidone	35	32.7
Aripiprazole	17	15.9
Paliperidone	20	18.7
Quetiapine	69	64.5
Clozapine	18	16.8
Amisulpride	20	18.7
Zuclopenthixol	20	18.7
Chlorpromazine	18	16.8

Toxicological analysis of postmortem body fluids revealed the presence of antidepressant agents in 21.7% (*n* = 23) of cases. Review of medical records showed that 36.4% of cases had a documented history of depot antipsychotic use. Distribution of antipsychotic use indicated that 50% (*n* = 53) of cases had a history of both first- and second-generation antipsychotic use, 33% (*n* = 35) had second-generation use only, 1.8% (*n* = 2) had first-generation use only, and 15% (*n* = 16) had no history of antipsychotic use. Clozapine use was documented in 17.7% (*n* = 19) of cases. Comparison of postmortem toxicology results with medical records indicated treatment adherence in 61.3% (*n* = 65) of cases.

Analysis of macroscopic organ findings according to antipsychotic use revealed that pleural petechial hemorrhage was significantly more frequent in antipsychotic users compared to non-users (*p* = 0.028). No significant differences were observed for other organ findings. No statistically significant associations were found between antipsychotic use and suicide or brain atrophy (*p* > 0.05). Mean brain weight was 1319 g in cases with a history of antipsychotic use and 1296 g in non-users, with no significant difference between groups. Similarly, no significant associations were observed between antipsychotic generation and brain atrophy.

No significant differences were found between antipsychotic use and yellowish liver appearance or presence of micronodular cirrhosis. Regarding event characteristics, although no overall group differences were observed, individuals with a history of combined antipsychotic use were significantly less likely to be found deceased compared to non-users (*p* = 0.003), whereas no significant differences were observed for other events (sudden illness, traumatic death). Evaluation of manner of death according to antipsychotic use in toxicology results revealed no significant associations (Table 6).

**Table 6:** Comparison of manner of death in cases with antipsychotics detected in toxicology

		Antipsychotic Use Detected in Toxicology				
		Present		Absent		
Manner of Death		***n***	***%***	***n***	***%***	***p***
Natural Death		*40*	*66.7*	*20*	*33.3*	0.243*
	Cardiovascular	24	68.6	11	48.8	
	Pneumonia	7	53.8	6	12.2	
	Infection	6	100.0	0	0.0	
	Cancer	2	50.0	2	50.0	
	Epilepsy	1	50.0	1	50.0	
Unnatural Death		*25*	*54.3*	*21*	*45.7*	0.099*
	Traffic Injury	0	0.0	1	100.0	
	Blunt Trauma	6	75.0	2	25.0	
	Sharp Force Injury	2	100.0	0	0.0	
	Asphyxia	6	85.7	1	14.3	
	Burn	1	50.0	1	50.0	
	Drowning	0	0.0	3	100.0	
	Poisoning	4	51.7	3	42.9	

** Analyses were performed using Fisher’s Exact chi-square test. *p* < 0.05 was considered significant

Among individuals with a history of clozapine use, no significant differences were observed in manner of death between groups. Cardiovascular disease was the most frequent natural cause of death among clozapine users (40%, *n* = 4). No significant association was observed between clozapine use and sudden cardiac death. Furthermore, no significant associations were observed between detection of antidepressants in toxicology or medical records and cause of death.

## Discussion

 Our study represents the first retrospective autopsy-based analysis in Türkiye reporting sudden death characteristics in patients with schizophrenia through both descriptive and comparative methods.

The majority of cases were male, with a mean age at death of approximately 50 ± 13.75 years. The predominance of males (70.1%) aligns with previous studies showing higher schizophrenia prevalence in men [19, 20]. The literature also indicates that male sex in schizophrenia is associated with increased mortality risk [21, 22]. Early disease onset, poor treatment adherence, substance use, and social isolation in men may contribute to this higher mortality [23]. The absence of a significant age difference between males and females is consistent with the chronic course of schizophrenia and the observation that deaths generally occur in middle age.

More than half of the deaths occurred as “found dead” cases, which may reflect the high prevalence of solitary living among schizophrenia patients and the frequent occurrence of unexpected deaths related to sudden cardiac or toxicological causes [24, 25]. Evidence suggests that the risk of sudden cardiac death in psychiatric patients is several times higher than in the general population [26, 27]. The nearly equal distribution of death locations in open and closed spaces reflects variability in patients’ living conditions and environmental influences on mortality. Similarly, previous studies report that a substantial proportion of sudden deaths in schizophrenia occur in home or public settings as “found dead” cases [28].

In our cohort, witnesses were present in a high proportion of death events, most commonly first-degree relatives (60.7%). This underscores the dependence of schizophrenia patients on family support and the potential role of timely intervention by relatives during acute health events [24, 29]. However, the occurrence of deaths in unwitnessed cases highlights the unpredictable and sudden nature of mortality in this population [14, 26].

Approximately half of the cases received medical intervention prior to death, indicating that many deaths occurred despite the availability of emergency care. Literature suggests that the frequency of sudden cardiac or toxicological deaths in schizophrenia is often related to delayed intervention or patients’ limited ability to communicate symptoms clearly [21, 28].

Autopsy findings for heart, lung, and brain weights were largely consistent with average values reported in the literature [24]. Myocardial lesions from previous ischemic injury (28.2%) and cardiac hypertrophy (19.4%) indicate known cardiovascular comorbidities in schizophrenia patients. These findings align with metabolic and cardiac risks associated with antipsychotic use [21, 23, 30]. Brain atrophy (19.6%) reflects the chronic neurodegenerative effects of schizophrenia and is frequently reported in older patients or those with longer disease duration [22]. Ischemic myocardial lesions and cardiac hypertrophy are consistent with well-known cardiometabolic and lifestyle-related risk factors in schizophrenia, including obesity, hypertension, diabetes, and smoking, whereas brain atrophy may be associated with long illness duration, potential neurotoxic effects, and comorbid metabolic disturbances.

 The presence of decomposition findings in 60% of discovered bodies underscores the importance of postmortem changes in relation to the timing of death detection and reporting. Additionally, petechial hemorrhage in the pleura (21.9%) and yellowish discoloration of the liver (36.8%) may indicate systemic effects or toxicological processes. These observations support the multifactorial nature of sudden death in schizophrenia patients, highlighting the roles of both cardiac and systemic pathologies [24, 28].

In our study, natural deaths were more frequent than unnatural deaths (56.1%), reinforcing the predominant role of cardiovascular disease in schizophrenia-related mortality. Among natural causes of death, cardiovascular events were the most common, accounting for 58.3% [24]. Previous studies also report that the risk of sudden cardiac death in schizophrenia patients is 2–3 times higher than in the general population, with additional risk factors including antipsychotic use, metabolic syndrome, hypertension, and obesity contributing to this increased risk [21, 23]. In a large autopsy-based study by Sun et al. including 391 patients with schizophrenia, 64.2% of deaths were attributed to natural causes, while approximately 33.2% resulted from unnatural causes such as accidents, suicides, and homicides, showing proportions comparable to those observed in the present study [16].

Natural causes such as pneumonia and other infections (21.7% and 10%), cancer (6.7%), and epilepsy (3.3%) are associated with immune system dysfunction, difficulties in accessing healthcare, and lifestyle factors in patients with schizophrenia [28, 31]. This highlights the vulnerability of these patients regarding monitoring chronic illnesses and timely medical intervention.

Unnatural deaths (43.9%) occurred through mechanisms such as blunt trauma (26.7%), poisoning, and asphyxiation. These findings reflect the elevated risk of suicide and accident-related mortality in schizophrenia patients. Similarly, the literature reports that a substantial proportion of sudden deaths in individuals with schizophrenia occur through traumatic or unnatural mechanisms [24, 26]. The cause of death could not be determined in 17 cases, illustrating the multifactorial nature of sudden death, which may remain indeterminate even with autopsy.

 No significant gender differences were observed regarding the manner of death, supporting the notion that sudden and traumatic deaths in schizophrenia patients can occur independently of gender [24, 26, 27]. In terms of clinical event type, natural deaths were significantly associated with illness events, whereas unnatural deaths were significantly associated with traumatic events (p<0.01), consistent with previously reported death mechanisms [21]. The significantly higher occurrence of skin decomposition in unnatural deaths suggests that postmortem changes may serve as an important indicator for early detection of traumatic events [28].

Regarding body mass index (BMI), the more frequent occurrence of cases with BMI below 25 among natural deaths suggests that low BMI may represent a risk factor, particularly under cardiovascular and metabolic stress. Literature indicates that low BMI in schizophrenia patients can increase mortality, sometimes in association with malnutrition or medication side effects [23, 32].

Postmortem toxicological examinations revealed the presence of antipsychotics in 61.3% of cases. The most frequently detected antipsychotic was quetiapine (64.5%), followed by risperidone (32.7%), paliperidone, amisulpride, and zuclopenthixol. Additionally, combined antipsychotic use was observed in 50% of cases. These findings indicate that polypharmacy is common in schizophrenia treatment and suggest that high doses or combination therapy may increase the risk of sudden death [24, 33]. The lack of significant differences in antidepressant use among death types suggests that these medications alone are not major determinants of sudden death risk based on current evidence.

When compared to an autopsy-based study conducted in China [14], our results show strong concordance. Wang and Li reported no gender differences in unexplained sudden deaths and higher mortality risk in young adults. They also noted common antipsychotic use and frequent polypharmacy in sudden unexpected death (SUD) cases, paralleling our toxicology findings.

These observations highlight the multifactorial nature of sudden death in schizophrenia patients, where cardiovascular, metabolic, and pharmacological risks interact. They also demonstrate the value of postmortem toxicological analyses in understanding death mechanisms and developing potential preventive strategies.

Identification is a critical aspect of postmortem evaluation. Tattoos were detected on external examination in 8.4% of cases, which may serve as a supportive tool in forensic identification [34, 35].

This study specifically evaluated antidepressant and antipsychotic use based on postmortem toxicology and medical records. Antidepressants were detected in 21.7% of cases, and 36.4% of cases had a history of depot antipsychotic use. Regarding antipsychotic generations, 50% of cases had a history of both first- and second-generation use, 33% had only second-generation use, 1.8% had only first-generation use, and 15% had no history of antipsychotic use. Clozapine use was recorded in 17.7% of cases. Comparison of postmortem toxicology with medical records indicated treatment adherence in 61.3% of cases. These findings suggest that antipsychotic use is prevalent among individuals with severe psychiatric disorders and that treatment adherence is generally achieved [21, 23].

In macroscopic organ evaluations, pleural petechial hemorrhage was significantly more frequent in antipsychotic users (p = 0.028). This finding may indicate potential effects of antipsychotic use on the vascular system and coagulation mechanisms. Literature reports that antipsychotics, particularly clozapine, may rarely cause hematologic side effects, including pleural hemorrhage [14, 28]. The absence of significant macroscopic differences in other organs suggests that systemic macroscopic changes due to antipsychotic use may be limited.

The effect of antipsychotic use on mortality risk is well-documented in the literature. In schizophrenia patients, the risk of sudden cardiac death may increase, particularly in association with antipsychotic use [24, 26]. Previous studies have shown that antipsychotics can affect potassium channel function, potentially leading to malignant arrhythmias [36, 37]. The findings of this study suggest that antipsychotic use may be associated with certain postmortem cardiopulmonary findings, while not significantly affecting general macroscopic organ changes. Additionally, factors such as gender-specific differences and treatment compliance emerge as important variables in assessing death risk and clinical course [38]. These results underscore the importance of considering pharmacological risks alongside clinical and sociodemographic characteristics in treatment planning for schizophrenia patients.

This study demonstrates that combined evaluation of postmortem toxicology and medical records provides a valuable approach to understanding the postmortem pathophysiological effects of antipsychotic and antidepressant use. The retrospective design and limited toxicology data in some cases constitute the main limitations. Future studies with larger sample sizes, molecular-level analyses, and gender-specific evaluations could more clearly elucidate the postmortem effects of antipsychotic use.

In the present study, antipsychotic use did not produce macroscopically significant effects on the brain, liver, or manner of death, nor was it significantly associated with suicide or brain atrophy (p > 0.05). The mean brain weight was 1319 g in individuals with a history of antipsychotic use and 1296 g in those without, with no significant difference between groups. No significant relationship was found between antipsychotic generation and brain atrophy. Similarly, no significant differences were observed in the yellowish appearance of the liver or the presence of micronodular cirrhosis.

Regarding event characteristics, no overall significant relationship was observed between antipsychotic use and being found dead, sudden illness, or traumatic death. However, individuals with a history of combined antipsychotic use had a significantly lower rate of being found dead (p = 0.003). No significant difference in manner of death was observed in individuals with a history of clozapine use, with cardiovascular disease being the most frequent natural cause of death (40%, n = 4). No significant association was found between clozapine use and sudden cardiac death. Similarly, no significant relationship was observed between detection of antidepressants and cause of death. Because combined antipsychotic treatment is often used in patients with a more severe clinical course or suboptimal therapeutic response, these individuals tend to remain under more frequent clinical contact and closer supervision. This increased level of monitoring may, in turn, reduce the likelihood of being alone at the time of death and increase the probability that the event is recognized earlier. In addition, depot antipsychotic formulations are commonly associated with improved treatment adherence and more regular clinical follow-up, which could contribute to earlier recognition of clinical deterioration or adverse outcomes.

These findings demonstrate that the postmortem pathophysiological effects of antipsychotic and antidepressant use are limited in terms of significant macroscopic changes in organs such as the brain and liver. Although previous studies have reported hematologic and cardiopulmonary side effects of antipsychotics [14, 24, 26, 28], the present data suggest that such effects may be limited at the macroscopic level. The observed reduction in the rate of being found dead among individuals with combined antipsychotic use highlights the complex interplay of treatment compliance and clinical condition. Similarly, the literature emphasizes the impact of treatment adherence and gender differences on mortality in schizophrenia patients [21, 23, 38]. The retrospective design of our study and its inclusion of only fatal cases constitute important limitations. A portion of the forensic autopsy cases had been found dead, and the postmortem interval may have complicated the accurate interpretation of histopathological and toxicological findings. In this study, the causes of death and postmortem findings in schizophrenia cases were analyzed. Deaths were predominantly due to natural causes, with cardiovascular disease playing a central role. Sudden and unnatural deaths appear to result from the interaction of multiple factors, including traumatic events, toxicological effects, and polypharmacy. Postmortem toxicology and macroscopic organ assessments indicate that antipsychotic and antidepressant use has a limited effect on postmortem pathophysiological changes. However, evaluating death risk based solely on medication use is insufficient, and factors such as gender, treatment adherence, and comorbidities must also be considered. The retrospective design of this study and the limited availability of toxicology data in some cases restrict the generalizability of these findings. Future research should involve larger sample sizes, molecular and biochemical analyses, and gender-specific evaluations to more clearly elucidate the postmortem effects of antipsychotic and antidepressant use and to inform clinical risk management.
